# Diallel Analysis of Chilli Pepper (*Capsicum annuum* L.) Genotypes for Morphological and Fruit Biochemical Traits

**DOI:** 10.3390/plants9010001

**Published:** 2019-12-18

**Authors:** Aiswarya C. S., Vijeth S, Sreelathakumary I, Prashant Kaushik

**Affiliations:** 1 Department of Vegetable Science, College of Agriculture, Kerala Agricultural University, Thrissur, Kerala 680656, India; aiswaryachandrasenannair2@gmail.com (A.C.S.); vijet129@gmail.com (V.S.); sreelatha.i@kau.in (S.I.); 2 Nagano University, 1088 Komaki, Ueda, Nagano 386-0031, Japan

**Keywords:** chilli pepper, combining ability, diallel, heterosis, capsaicin, oleoresin, ascorbic acid, colour

## Abstract

Chilli pepper is commercially cultivated as a spice and is also used for the extraction of a colouring agent. Here, we performed a diallel genetic study involving five chilli pepper varieties. Parents and their hybrid were evaluated for fifteen morphological and five biochemical traits over two crop seasons under open field conditions. Variation was recorded for all of the studied traits. Similarly, significant values for general combining ability (GCA) and specific combining ability (SCA) variance were obtained for all of the traits. The ratio of σ^2^ SCA/σ^2^ GCA indicates that non-additive gene effects were predominant for all the studied traits except for fruits plant^−1^. Based on SCA effects, cross combinations P2 × P5, and P4 × P5 were determined excellent for flesh thickness, yield components and vitamin C. These hybrids are recommended for multilocation testing to assess their suitability for commercial cultivation. Overall, this work presents useful information regarding the genetics of important morphological and biochemical traits in chilli pepper.

## 1. Introduction

Chilli pepper (*Capsicum annuum* L., 2n = 2 × = 24), belongs to the family Solanaceae. Chilli is an important spice crop with high therapeutic value of its biochemical constituents [1]. Among five cultivated species, *Capsicum annuum* L. is the most extensively cultivated. Chilli possesses antioxidant therefore nutritional properties, and it is being regularly used in medicine and pharmaceutical industries [2,3]. Globally, chillies occupy an area of 2.75 million ha with a production of 53.91 million tonnes and productivity of 32.13 tonnes ha^−1^ [4]. Pungency in chilli is because of capsaicinoids, a group of 15 different alkaloids. Capsaicin and dihydrocapsaicin are the major alkaloids which contribute up to 90% of the total capsaicinoids [5]. The red colour in chilli pepper is mainly due to the significant presence of colouring compounds, namely capsanthin and capsorubin, collectively known as oleoresin. It is an oil-soluble extract and is primarily being used as a colouring and flavouring agent in food products [6,7]. Oleoresin is extensively used in meat processing, beverage, pharmaceutical and cosmetic industries as a substitute for the synthetic colour used in food and cosmetic industries [8,9].

Ascorbic acid is an antioxidant that plays a vital role in human nutrition and body functioning. Assessing the quality of chilli pepper colour value is among the principal criteria. Ascorbic acid is actively involved in neutralising free radicals, iron assimilation, wound healing process, and protecting the skin from viral and bacterial infection by building collagen in the skin [10,11]. Similarly, according to recent reports, capsaicin induces vigorous anticancer activity, particularly against prostate cancer [12,13]. In chilli, the hybrid seed production mainly relies on the hand emasculation. Although there are several reports of employing cytoplasmic male sterility (CMS) and genetic male sterility (GMS) in the chilli pepper, the system is still in the early phase of development. Moreover, when employing GMS for hybrid seed production, the segregation into male sterile and male fertile plants is commonly noticed, as is the difficulty of rouging plants with fertile pollens [14].

Moreover, the genetics of chilli pepper are not well studied as compared to other members of Solanaceae [15,16,17]. Therefore, many concentrated efforts are necessary to develop improved quality hybrids with high yield. The ability of parents to perform better in a hybrid combination depends on the genes, which cannot be merely adjudged by per se performance of the parents [18]. For the selection of parents, combining ability is considered as the essential criterion [19]. Chilli offers much scope of improvement with respect to quality and yield traits through heterosis breeding, which can further be utilised for the development of desirable recombinants [20]. The diallel mating design is commonly used by the plant breeders to determine the bases of inheritance of quantitative traits Among the various schemes of a diallel mating design, the halfway diallel cross (Method II, Model I) is more manageable, as it includes one-directional crosses as compared to the doubled crosses in a full diallel mating scheme [21]. A diallel study is a useful tool for preliminary evaluation of genetic stock for use in hybridisation programme, and to identify superior general as well as specific combiners [22]. Therefore, the present investigation assessed the extent of heterosis in desirable direction and gene action necessary for quality parameters to identify good general and specific combiners and to design the breeding strategy for the genetic improvement of yield and quality traits.

## 2. Results

### 2.1. Analysis of Variance for the Experimental Design

Mean squares owing to parents, hybrids and parents versus hybrids were highly significant for all traits (Table 1). Similarly, the pooled analysis of variance over two seasons for morphological (15) and fruit biochemical (5) traits showed that mean squares due to general combining ability (GCA) and specific combining ability (SCA) were highly significant for all studied traits (*p* ≤ 0.01) (Table 2). The results also indicate the involvement of both additive and non-additive genetic variance in governing the expression of the studied traits. The magnitude of SCAs were high for fruits plant^−1^, seeds fruit^−1^, green fruit yield plant^−1^, dry fruit yield plant^−1^, yield plot^−1^, capsaicin and ascorbic acid. For the remaining traits, the magnitude of GCAs were maximum (Table 2). The values of σ^2^ SCA were higher for all the studied traits except for fruits plant^−1^. Non-additive gene effects played a significant role in the inheritance of these traits (Table 2). For fruits plant^−1^, the value of σ^2^ GCA was highest, indicating the predominance of additive gene effects (Table 2). %The ratio of σ^2^ SCA/σ^2^ GCA suggests the nature of inheritance for a particular trait and its values were larger (>1) for all of the traits except for fruits plant^−1^ (Table 2).

### 2.2. GCA and SCA Effects 

The combining ability analysis showed that parent P5 expressed high GCA effects for fruits plant^−1^, fruit weight and green fruit yield plant^−1^ (Table 3). Besides, it also exhibited significant and positive GCA values for flesh thickness, flesh to seed ratio, driage, oleoresin and colour value (Table 3). Parents P1, P2 and P3 had significant negative values for fruit traits and yield components (Table 3). Parent P3 expressed high positive considerable GCA effects for days to first flower and days to first harvest (Table 3). Parent P2 had a significant positive value for ascorbic acid while this parent had negative values for yield components. For fruit length, flesh thickness seeds plant^−1^ and capsaicin, parent P1 expressed high GCA effects (Table 3). 

The SCA values for the cross combinations are presented in Table 4. The cross combination P1 × P2 showed high significant positive SCA effects for plant height, primary branches plant^−1^, fruit girth and ascorbic acid content (Table 4). The hybrid combination P2 × P3 showed the highest SCA effects for days to first flower and days to first harvest (Table 4). The cross combination P4 × P5 exhibited high positive SCA effects for yield plot^−1^ (Table 4). The SCA effects were high and positively significant in hybrid P2 × P5 for fruit length, fruit weight and seeds fruit^−1^. The hybrid P1 × P3 showed maximum SCA effects for capsaicin (Table 4).

### 2.3. Heterosis

A significant amount of mid parent heterosis (MPH) was noticed for all studied traits (Table 5). The cross combination P4 × P5 showed the maximum positive heterosis for the yield plot^−1^ (142%) and fruits plant^−1^ (103%) (Table 5). The highest MPH fruit weight was determined in the cross combination P3 × P5 (58%). For capsaicin content and colour value, the cross combination P1 × P3 exhibited the maximum MPH of 82% and 18%, respectively (Table 5). Similarly, in the case of better parent heterosis (BPH), significant and positive heterosis values were displayed by most of the hybrids (Table 5). Except for the traits days to first flower and days to first harvest for both the traits, none of the hybrid combinations showed positive MPH or BPH values (Table 5). The cross combination P4 × P5 displayed maximum BPH for yield and yield contributing traits and the hybrid cross combination P1 × P3 for the biochemical traits capsaicin and colour value (Table 5).

### 2.4. Correlations

**Figure 1:**
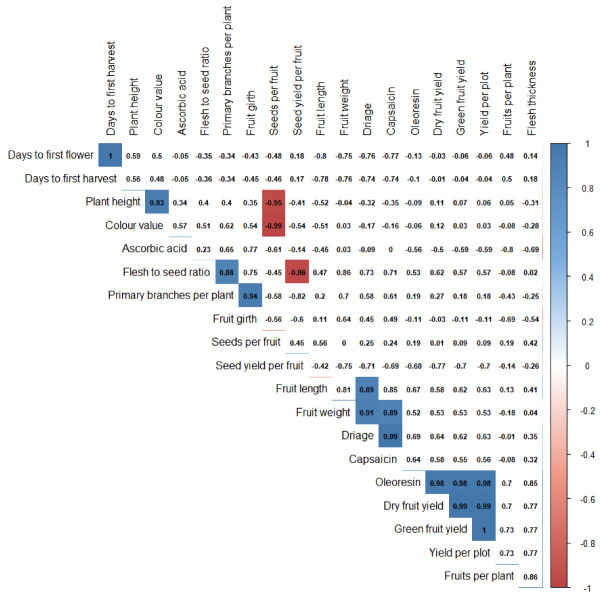
Pearson’s correlation coefficients of parental lines of chilli pepper with significant values *p* < 0.05 highlighted.

**Figure 2:**
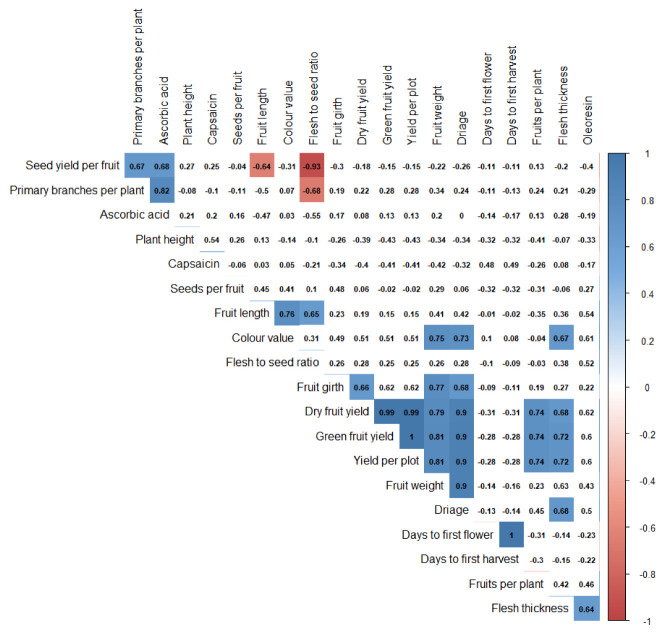
Pearson’s correlation coefficients of hybrids of chilli pepper with significant values *p* < 0.05 highlighted.

Among the parents, seventeen correlations were observed to be significant (*p* < 0.05) (Figure 1). There were three absolute negative correlations (≥0.9) and the remaining were positive correlations (Figure 1). Seeds per fruit were negatively correlated with the plant height and colour value. Yield per plot was absolutely correlated with dry fruit weight, green fruit yield, and oleoresin content (Figure 1). In parents, capsaicin content was significantly correlated with fruit weight and driage (Figure 1). In the case of hybrids, thirty-three correlations were determined to be significant (*p* < 0.05) (Figure 2). There were in total eight absolute correlations (Figure 2). Among hybrids, the flesh to seed ratio was determined to be negatively correlated with the seed yield per fruit and primary branches per plant. Interestingly, among hybrids yield per plot was determined to be correlated with fruit weight, flesh thickness and driage (Figure 2). Concerning hybrids, the colour value was correlated with fruit weight and driage. In addition, the capsaicin content was not correlated with any other trait (Figure 2).

## 3. Discussion

Chilli pepper improvement is crucial for securing the higher yield of this crop. We found highly significant differences for all studied traits. Significant differences among chilli pepper genotypes were also reported [23,24,25,26,27]. In the present study, the GCA and SCA variance was significant (*p* ≤ 0.01) for all studied traits. This suggested the influence of both additive and non-additive gene effects on hybrid performance. The inheritance of a particular trait could be identified based on the ratio of GCA and SCA variance [28]. The non-additive gene effects played a significant role than additive effects in all studied traits except fruits plant^−1^. Previously, in chilli pepper, Hasanuzzaman et al. [29] reported the non-additive genetic control of plant height. Bhutia et al. [30] also observed non-additive gene effects for primary branches plant^−1^, days to first flower, fruit length, fruit girth and seeds fruit^−1^.

Heterosis breeding provides an opportunity to increase productivity in chilli pepper. The primary objective of heterosis is to achieve high yield potential and good quality aspects of the crop plants [31]. Commercial hybrids are becoming more popular than the open-pollinated cultivars because of superiority in yield and quality traits. Hybrids are becoming popular in many crops as they give an opportunity to utilise the synergistic effect of a genetic combination [32]. For a systematic breeding program, it is essential to identify the parents as well as crosses to bring genetic improvement in economic character. The magnitude of heterosis depends on the genetic diversity existing between the parents [33]. In a crop such as chilli pepper, where there is evidence for polygenic action determining the yield and the yield components, the choice of parents must be based on refined biometrical techniques. The value of genotypes depends on the ability to produce superior hybrids in combination with other genotypes [34].

The traits with higher GCA/SCA values possess a chiefly additive genetic control. In contrast, the traits with low GCA/SCA ration have a predominantly non-additive genetic control [35,36]. In our study, the predominance of SCA could be due to less diversity among the parents. This infers that breeding for yield and its contributing traits will not only require parents holding higher GCA values, in addition to specific hybrid combinations that will result in the expression of the trait. The present results are also in accordance with the outcomes of Gopalakrishna et al. [37], Shukla et al. [38] and Bhutia et al. [30]. However, additive gene effects were also reported for this trait [23,39]. For fruit weight, non-additive gene effects were observed in this study. Contrary to this, Rego et al. [40] observed additive gene effects controlling this trait. In the present study, capsaicin was controlled by non-additive gene effects; previous findings showed that this trait could be governed by either additive gene effects [41] or by non-additive effects [42]. In our study, a high magnitude of non-additive gene effects were expressed for green fruit yield plant^−1^, and this result was in accordance with a previous report [38]. However, the opposite was reported by Rego et al. [40]. Non-additive gene effects are contributing to capsaicin, and it was supported by the findings of Butcher et al. [27].

The cross combination P2 × P5 was good specific combiner for fruit length, colour value, oleoresin and seed fruit^−1^. The hybrid P4 × P5 expressed high SCA values for fruit weight, green fruit yield plant^−1^, driage and seed yield fruit^−1^. Similar results were also obtained by Payakhapaab et al. [43]. For days to first flower and fruits plant^−1^, the cross combination P3 × P4 exhibited high SCA effects. Good general combiners for fruits plant^−1^ was reported by Perez-Grajales et al. [44]. Concerning heterosis, positive heterosis was observed for eight traits and negative heterosis for two traits. The positive and negative heterosis was determined for ten characters. Heterosis over mid-parent has also been reported for fruit traits and yield components in chilli peppers, as well as in other members of the Solanaceae [15,17,30].

Overall, in the present study, non-additive gene effects are predominant for all the studied traits except for fruits plant^−1^ and these traits can be improved through heterosis breeding by exploiting hybrid vigour. Parental P5 was a good general combiner for fruit traits and yield components, followed by parent P4. Parents P5 and P4 were also good general combiners for colour value and oleoresin. Parent P1 was a good general combiner for capsaicin, whereas parent P2 for vitamin C. Based on SCA effects cross combination, P4 × P5 was an excellent specific combiner for flesh thickness, yield components and vitamin C. The hybrid P2 × P5 was good general combiners for oleoresin and colour value, whereas the hybrid P1 × P2 was the best general combiner for vitamin C. These hybrids can be used further in segregation generation analysis, to identify superior stable segregants with high yield and superior quality. 

## 4. Materials and Methods 

### 4.1. Plant Material, Design and Layout

The materials for the study comprised five parents (Table 6). The five parents were selfed, and they were crossed in a half diallel method to obtain ten F1 hybrids. The hybrids were produced via hand emasculation. Firstly, the well-developed flower-buds likely to open the next morning were emasculated during evening hours and bagged. Later, the buds were pollinated with the male parents (between 08:00 and 10:00) and subsequently bagged with the labelled paper bags. The mature crossed fruits were harvested, and the seeds were collected separately from each cross. For maintenance of parental lines, flower buds of different parents were selfed by bagging the individual buds and properly tagged and later the seeds were collected from the mature fruits accordingly. The experiment was laid out in randomised complete block design consisting of 15 treatments and three replications for two seasons, viz. May 2015 to September 2015 (first season) and October 2015 to February 2016 (second season). Thirty-day-old seedlings having 8–10 cm height were transplanted into the main field at a spacing of 45 cm × 45 cm. The crop received timely management practices as per package of practices recommendations of Kerala Agricultural University (coordinates at 10.54° N, 76.28° E) Thrissur, Kerala, India [45]. The weather information during the crop seasons is provided in Table 7. Further, the soil was sandy loam with a soil pH of 5.8.

### 4.2. Morphological Traits

There were twenty plants in each replication, and the morphological traits were determined from the sample of five randomly selected plants from the three replications. Plant height (cm) and the number of primary branches per plant were recorded at the time of peak harvest. Days to the first flower was estimated based on the average date of transplanting to the first flowering. Days to first harvest (earliness) were determined as the number of days from the date of transplanting to the first fruit harvest.

The total number of fruits produced per plant from all of the plants was counted, and the average was worked out to estimate the total number of fruits per plant. Ten fruits were selected at random from the observational plants to determine the fruit length (cm) and fruit girth (cm). Fruit Weight (g) was recorded as the mean of the representative sample of fruits. Flesh Thickness (mm) was determined as the thickness of fruit pericarp. Flesh to seed ratio was estimated as the ratio of flesh weight/seed weight of fruit from the sample of twenty representative fruits. Seeds per fruit were counted in five fruits, and the average was taken as seeds per plant. Green fruit and dry fruit yield per plant (g) were recorded, an average was worked out and expressed in grams per plant. Yield (kg) was estimated on the per plot basis by determining the fruit harvest of each plot. 

### 4.3. Fruit Biochemical Traits

Driage percentage was calculated as the weight change before and after oven drying at 70 °C based on the formula: 100 × (weight of dried fruit/weight of fresh fruit). For the capsaicin content (%), the pungent principle (capsaicin) reacts with Folin–Dennis reagent to give a blue coloured complex, which is estimated colourimetrically [46]. In short, an aliquot of 1 mL was pipetted into 100 mL conical flask, 25 mL of Folin–Dennis reagent were added and then it was kept for about 30 min. Afterwards, sodium carbonate solution was added with distilled water to make a volume of 100 mL. Next, the optical density was determined at 725 nm with a spectrophotometer (Jenway, Essex, UK). Oleoresin (%) was determined with the help of a Soxhlet’s apparatus (HMSOX-250, Illinois, United States) and acetone as a solvent. Oleoresin was calculated as the per cent of the weight of oleoresin to the weight of the sample. Ascorbic acid (mg 100^−1^ g of fresh fruit weight) content of fruit was estimated by 2,6-dichlorophenol indophenol dye method [47]. Firstly, a stock solution was prepared with ascorbic acid (100 mg) in 100 mL of 4% oxalic acid. Then, 10 mL of this stock solution were diluted to 100 mL, with 4% oxalic acid. Forty-two milligrams sodium bicarbonate were dissolved in a small volume of distilled water. Fifty-two milligrams of 2,6-dichlorophenol indophenol (dye) were added into this and made up to 200 mL with distilled water. Then, 5 mL of the working standard solution were pipetted into a 100 mL conical flask and 10 mL of 4% oxalic acid were added. The endpoint of the titration is the appearance of a pink colour, which persisted for at least 5 s. Colour value was determined according to the AOAC procedure [48]. Briefly, red ripe fruits were dried, and the stalk and seeds were removed before powdering. Then, 0.1 g of ground chilli powder was transferred into a 250 mL flask with 100 mL isopropanol and kept overnight at room temperature. The contents were filtered through a Whatman No. 42 filter paper. The first 10 mL were discarded, and 25 mL of the filtrate were pipetted into a volumetric flask and diluted with isopropanol. At 450 nm absorbance with a spectrophotometer (Jenway, Essex, UK), the colour was determined using isopropanol as blank. Standard colour solution was prepared by dissolving 0.5 mg per mL of reagent grade potassium dichromate into 1.8 M sulphuric acid.

### 4.4. Data Analysis 

The analysis for general and specific combining ability and their effects were computed by Method II (parents plus one set of crosses), Model 1 (fixed-effect model), as suggested by Griffing [21], using the AGD-R [49]. Mid parent heterosis was determined as the percentage of increase or decrease of F_1_ hybrids over mid-parent. Pearson’s linear correlation coefficients were estimated using the Statgraphics Centurion XVI software program. Mid-parent heterosis was determined based on the formula: 100 × ((F_1_ − MP) / MP), where F_1_ is the hybrid mean and MP is the mean of the parents. The better parent heterosis was estimated as: 100 × ((F_1_ − BP) / BP), where F_1_ is the hybrid mean and BP is the mean of the better parent.

## Figures and Tables

**Table 1:** Analysis of variance of parents, hybrids and parents vs. hybrids.

Source of Variation	Replicates	Treatments	Parents	Hybrids	Parents vs. Hybrids	Error
d.f	2	14	4	9		32
Plant height (cm)	31.33	218.16 **	575.20 **	59.96 **	153.66 **	14.18
Primary branches plant-1	0.04	1.52 **	1.86 **	1.69 **	0.36 **	0.02
Days to first flower	0.05	13.08 **	0.81 **	4.29 **	73.91 **	0.02
Days to first harvest	0.01	12.80 **	0.56 **	4.29 **	71.49 **	0.02
Fruits plant-1	49.85	4565.15 **	378.71 **	4191.18 **	24,308.19 **	32.25
Fruit length (cm)	0.04	6.96 **	3.92 **	2.28 **	26.94 **	0.41
Fruit girth (cm)	0.32	1.88 **	0.76 **	0.50 **	0.11 *	0.02
Fruit weight (g)	0.19	30.77 **	2.51 **	10.47 **	28.97 **	0.15
Flesh thickness (mm)	0.98	0.66 **	0.34 **	0.25 **	0.07 **	2.30
Flesh to seed ratio	0	14.37 **	11.50 **	10.00 **	1.07 **	0
Seeds fruit-1	1.19	1932.53 **	610.69 **	589.25 **	13,138.21 **	10.49
Green fruit yield plant-1 (g)	594.46	132,993.00 **	15,711.40 **	90,901.51 **	1,085,263.25 **	901.3
Dry fruit yield plant-1 (g)	14.82	3568.28 **	418.91 **	2442.97 **	29,087.32 **	24.2
Yield plot-1 (kg)	0.49	110.28 **	13.03 **	75.37 **	900.22 **	0.75
Driage (%)	0.07	25.87 **	6.07 **	27.23 **	0.53	0.19
Seed yield fruit-1 (g)	0.5	0.11 **	0.08 **	0.09 **	0.00 **	2.0
Capsaicin (%)	0	0.06 **	0.00 **	0.00 **	0.88 **	1.37
Oleoresin (%)	0.31	21.11 **	5.49 **	26.45 **	63.13 **	0.07
Ascorbic acid (mg/100g)	0.14	332.59 **	8.40 **	232.22 **	2270.04 **	0.66
Colour (ASTA units)	0.18	1154.62 **	1245.60 **	913.54 **	1537.60 **	0.44

** and * indicate significant at *p* < 0.01 and *p* < 0.05, respectively.

**Table 2:** Pooled Analysis of variance over two crop seasons for general combining ability (GCA) and specific combining ability (SCA) for the fifteen morphological and five biochemical descriptors in chilli pepper.

Source	Replications	Genotypes	Error	GCA	SCA	Error	σ^2^ GCA	σ^2^ SCA	σ^2^ SCA/σ^2^ GCA
Df	2	14	28	4	10	28			
Plant height (cm)	67.78 **	209.62 **	11.72	73.16 **	68.54 **	3.9	1.31	1.47	1.12
Primary branches per plant	0.05	2.02 **	0.015	0.977 **	0.55 **	0.0037	0.12	0.54	4.5
Days to first flower	0.1 **	7.51 **	0.02	1.32 **	2.97 **	0.0063	0.46	2.82	6.13
Days to first harvest	0.125 **	7.2 **	0.02	1.304 **	2.83 **	0.0064	0.43	2.82	6.55
Fruits plant^−1^	291.56 **	3166.03 **	53.29	697.29 **	1198.56 **	17.75	1432.15	1180.8	0.82
Fruit length (cm)	3.08	4.61 **	0.02	2.23 **	1.25 **	0.0049	0.28	1.2	4.28
Fruit girth (cm)	0.08	0.51 **	0.01	0.173 **	0.16 **	0.0027	0.03	0.16	5.33
Fruit weight (g)	0.095 **	8.65 **	0.01	4.285 **	2.31 **	0.001	0.56	2.31	4.12
Flesh thickness (mm)	0.01	0.24 **	0.01	0.143 **	0.054 **	0.001	0.02	0.05	2.5
Flesh to seed ratio	0.01	10.00 **	0.01	6.8 **	1.94 **	0.005	1.38	1.94	1.4
Seeds per fruit	85.47	1447.70 **	1.56	275.47 **	565.4 **	0.732	82.83	564.88	6.81
Green fruit yield per plant (g)	6547.43 **	147,164.15 **	3941.25	16,235.04 **	54,302.00 **	1313.74	5247.36	52,988.35	10.09
Dry fruit yield per plant (g)	131.03 **	3494.14 **	24.89	933.11 **	1257.35 **	8.29	92.63	1249.05	13.48
Yield per plot (kg)	5.38 **	122.04 **	32.65	29.8 **	45.02 **	1.08	4.35	43.94	10.1
Driage (%)	5.36 **	16.53 **	0.075	10.87 **	3.36 **	0.025	2.14	3.33	1.55
Seed yield per fruit (g)	0.24 **	0.08 **	0.01	0.230 **	0.049 **	0.001	0.007	0.018	2.57
Capsaicin (%)	0.01	0.065 **	0.01	0.005 **	0.0165 **	0.001	0.008	0.03	3.75
Oleoresin (%)	5.01	23.085 **	0.01	9.23 **	7.07 **	0.0065	0.61	7.07	11.59
Ascorbic acid (mg/100g)	18.24 **	3422.15 **	0.26	30.79 **	147.34 **	0.18	33.25	147.25	4.42
Colour value (ASTA units)	220.17 *	1051.07 **	0.18	438.72 **	0.497 **	0.058	35.34	314.94	8.91

** and * indicate significant at *p* < 0.01 and *p* < 0.05, respectively.

**Table 3:** General combining ability (GCA) effects for fifteen morphological and five biochemical traits of chilli pepper.

Traits	P1	P2	P3	P4	P5
Plant height (cm)	−4.6 **	−1.4 *	3.17 **	2.87 **	0.13
Primary branches plant^−1^	−0.49 **	0.14 **	−0.25 **	0.16 **	−0.43 **
Days to first flower	−0.075 **	−0.15 **	0.64 **	−0.525 **	−0.085 **
Days to first harvest	−0.09 **	−0.155 **	0.63 **	−0.515 **	−0.1 **
Fruits plant^−1^	−8.77 **	−8.05 **	8.44 **	−3.87 **	9.09 **
Fruit length (cm)	1.46 **	−0.40 **	−0.64 **	−0.06 **	0.37 **
Fruit girth (cm)	0.04 *	0.48 **	0.15 **	0.05 **	0.25 **
Fruit weight (g)	−0.24 **	−0.6 **	−0.79 **	0.09 **	1.14 **
Flesh thickness (mm)	0.041 **	−0.055 **	−0.037 **	−0.165 **	0.205 **
Flesh to seed ratio	0.445 **	−1.16 **	−0.895 **	0.475 **	1.125 **
Seeds fruit-1	9.74 **	2.14 **	−4.9 **	−6.09 **	−1.58 **
Green fruit yield plant^−1^ (g)	−20.07	−76.84 **	−14.49	5.83	115.56 **
Dry fruit yield plant^−1^ (g)	−3.78 **	−12.11 **	−1.96 *	2.09 *	18.71 **
Yield plot^−1^ (kg)	−0.575 *	−2.20 **	−0.41 *	0.43 *	3.32 **
Driage (%)	0.085	−0.915 **	−0.115 *	0.06	2.01 **
Seed yield fruit^−1^ (g)	−0.04 *	0.085 **	0.061 **	0.003 *	0.115 **
Capsaicin (%)	0.0045 **	0.001 *	0.0015 *	−0.005 **	−0.002 **
Oleoresin (%)	0.035 **	−1.51 **	−0.28 **	0.10 **	1.67 **
Ascorbic acid (mg/100g)	−2.6 **	3.03 **	0.12	−1.09 **	0.54 **
Colour value (ASTA units)	−7.54 **	−5.00 **	−1.06 **	0.69 **	12.92 **

** and * indicate significant at *p* < 0.01 and *p* < 0.05, respectively.

**Table 4:** Specific combining ability (SCA) effects exhibited by ten F_1_ hybrids for the fifteen morphological and five biochemical traits of chilli pepper.

Parents	P1 × P2	P1 × P3	P1 × P4	P1 × P5	P2 × P3	P2 × P4	P2 × P5	P3 × P4	P3 × P5	P4 × P5
Plant height (cm)	12.02 **	2.64 **	7.74 **	0.74	2.54 *	−1.18	7.31 **	−2.28	−10.155 **	−4.52 **
Primary branches plant^−1^	0.87 **	0.4 **	−0.22 **	−0.58 **	0.68 **	−0.19 **	0.18 **	−0.66 **	0.87 **	0.32 **
Days to first flower	−1.52 **	0.30 **	−0.59 **	0.075	0.445 **	−0.995 **	−1.98 **	−2.18 **	−0.77 **	−0.66 **
Days to first harvest	−1.52 **	0.30 **	−0.59 **	0.075	0.445 **	−0.995 **	−1.98 **	−2.18 **	−0.77 **	−0.66 **
Fruits plant^−1^	12.26 **	−5.33 **	−7.95 **	−10.07 **	8.80 **	−9.91 **	17.05 **	44.35 **	23.12 **	38.18 **
Fruit length (cm)	−0.40 **	0.57 **	1.07 **	0.77 **	0.82 **	0.51 **	1.52 **	0.64 **	−0.1 *	−0.05
Fruit girth (cm)	0.55 **	−0.06 **	−0.065 **	0.13 **	−0.17 **	−0.56 **	0.24 **	0.07 *	0.56 **	−0.30 **
Fruit weight (g)	1.19 **	−0.31 **	0.92 **	0.075 **	−0.275 **	−0.63 **	1.53 **	−0.77 **	2.15 **	1.44 **
Flesh thickness (mm)	−0.095 **	−0.04 **	0.095 **	−0.155 **	−0.025 **	0.075 **	0.15 **	0.15 **	−0.24 **	0.46 **
Flesh to seed ratio	1.035 **	0.53 **	−0.47 **	1.48 **	0.1 **	−0.79 **	0.075 **	1.45 **	2.22 **	−0.70 **
Seeds fruit^−1^	8.20 **	16.32 **	15.92 **	1.20 **	−9.76 **	12.96 **	33.81 **	18.90 **	21.32 **	−0.62
Green fruit yield plant^−1^ (g)	115.85 **	−25.91 **	134.48 **	54.53 **	44.17 **	−35.31	182.30 **	172.20 **	207.80 **	275.53 **
Dry fruit yield plant^−1^ (g)	22.31 **	−3.95	23.17 **	8.24 **	6.85 **	6.40 **	29.41 **	31.85 **	29.83 **	34.09 **
Yield plot^−1^ (kg)	3.97 **	−0.73	3.85 **	1.56 *	1.27	−1.01	5.24 **	4.96 **	5.97 **	7.93 **
Driage (%)	−0.62 **	−2.32 **	1.42 **	−0.2 *	0.315 **	−2.83 **	1.38 **	−0.62 **	2.14 **	1.48 **
Seed yield fruit-1 (g)	−0.03 **	0.005 *	0.04 **	−0.03 **	−0.02 **	0.02 **	0.06 **	0.09 **	−0.17 **	0.12 **
Capsaicin (%)	0.07 **	0.115 **	0.10 **	0.07 **	0.15 **	0.07 **	0.11 **	0.09 **	0.06 **	0.09 **
Oleoresin (%)	−1.30 **	2.27 **	0.91 **	1.35 **	−2.21 **	0.52 **	3.82 **	3.23 **	−0.42 **	1.63 **
Ascorbic acid (mg/100g)	13.90 **	12.4 **	−1.53 **	−10.68 **	3.09 **	1.98 **	11.83 **	−0.59 **	8.24 **	13.47 **
Colour value (ASTA units)	−0.86 **	16.42 **	21.50 **	13.77 **	−3.10 **	−9.15 **	25.63 **	−20.27 **	−2.56 **	−0.29 *

** and * indicate significant at *p* < 0.01 and *p* < 0.05, respectively.

**Table 5:** Mid parent heterosis (MPH) and better parent heterosis (BPH) values (%) for the morphological and biochemical traits in the chilli pepper hybrids.

Traits	MPH/BPH	P1 × P2	P1 × P3	P1 × P4	P1 × P5	P2 × P3	P2 × P4	P2 × P5	P3 × P4	P3 × P5	P4 × P5
Plant height (cm)	MPH	36.62 **	8.68 *	18.87 **	7.01	7.73 *	5.98	15.37 **	−6.52 *	−17.05 **	−8.04 *
	BPH	29.14 **	−10.45 **	−0.24	−8.59 *	−6.94	−6.63	3.59	−8.62 *	−20.59 **	−9.98 *
Primary branches per plant	MPH	64.56 **	45.21 **	0	−13.73 **	72.15 **	27.36 **	31.48 **	−6.00 *	45.10 **	13.18 **
	BPH	52.94 **	45.21 **	−21.26 **	−32.82 **	60.00 **	6.30 **	8.40 **	−25.98 **	12.98 **	11.45 **
Days to first flower	MPH	−9.73 **	−0.6	−8.33 **	−4.26 **	−2.24 **	−10.14 **	−12.85 **	−12.96 **	−7.80 **	−9.31 **
	BPH	−11.03 **	−3.49 **	−9.00 **	−5.53 **	−3.72 **	−10.79 **	−12.95 **	−14.88 **	−9.30 **	−9.86 **
Days to first harvest	MPH	−5.75 **	−0.36	−4.48 **	−2.52 **	−1.34 **	−5.87 **	−7.63 **	−6.88 **	−4.66 **	−5.38 **
	BPH	−6.55 **	−2.10 **	−4.76 **	−3.28 **	−2.24 **	−6.41 **	−7.69 **	−8.25 **	−5.59 **	−5.85 **
Fruits per plant	MPH	40.67 **	16.51 *	64.55 **	48.70 **	52.26 **	89.79 **	76.94 **	71.74 **	46.34 **	103.12 **
	BPH	24.14 *	11.54	34.25 **	43.34 **	29.39 **	73.26 **	61.40 **	35.48 **	35.27 **	70.69 **
Fruit length (cm)	MPH	8.05 **	16.09 **	20.46 **	16.89 **	23.53 **	18.51 **	30.20 **	21.54 **	14.91 **	12.48 **
	BPH	−3.95 **	1.35 *	11.13 **	12.49 **	21.01 **	13.80 **	19.87 **	14.43 **	3.82 **	7.64 **
Fruit girth (cm)	MPH	22.24 **	5.00 *	−4.91 *	14.52 **	−3.16	−19.56 *	10.04 **	−2.81	20.02 **	−7.70 **
	BPH	18.02 **	4.31	−13.73 **	5.68 *	−7.10 **	−24.59 *	5.00 *	−12.34 **	10.09 **	−9.40 **
Fruit weight (g)	MPH	45.50 **	10.16	22.00 **	26.40 **	7.53	4.5	56.18 **	12.37	58.89 **	42.93 **
	BPH	23.47 **	−2.01	22.00 **	25.42 **	1.89	−11.32	31.69 **	−0.05	40.38 **	41.82 **
Flesh thickness (mm)	MPH	−5.22 **	−2.44 **	16.23 **	−3.58 **	0.23	15.23 **	9.05 **	24.36 **	−6.11 **	29.42 **
	BPH	−11.03 **	−6.21 **	−7.72 **	−5.56 **	−2.24 **	−3.77 **	0.4	1.79*	−11.51 **	1.19
Flesh to seed ratio	MPH	44.46 **	33.44 **	4.60 **	44.77 **	7.50 **	−13.72 **	0.98	26.08 **	−32.73 **	−11.09 **
	BPH	22.80 **	18.22 **	−10.21 **	16.59 **	2.50 *	−35.21 **	−27.92 **	−2.09 **	−50.51 **	−17.67 **
Seeds per fruit	MPH	27.54 **	39.46 **	40.30 **	26.15 **	13.10 **	40.99 **	65.50 **	53.21 **	55.64 **	33.00 **
	BPH	18.82 **	21.52 **	18.35 **	9.47 **	5.21 **	26.60 **	53.26 **	47.46 **	54.88 **	28.61 **
Green fruit yield per plant (g)	MPH	91.13 **	29.48 *	102.18 **	70.33 **	66.86 **	89.75 **	113.31 **	99.72 **	90.86 **	142.70 **
	BPH	52.38 **	26.41	73.27 **	65.57 **	35.50 *	73.87 **	66.51 **	74.70 **	81.25 **	103.14 **
Dry fruit yield per plant (g)	MPH	91.23 **	29.86 **	102.79 **	55.16 **	64.66 **	86.82 **	90.77 **	104.17 **	64.75 **	88.76 **
	BPH	52.95 **	27.62 **	73.59 **	40.38 **	33.47 **	72.08 **	41.69 **	77.38 **	46.75 **	48.78 **
Yield per plot (kg)	MPH	91.20 **	29.65 *	101.91 **	70.35 **	67.14 **	89.81 **	113.35 **	100.00 **	91.04 **	142.76 **
	BPH	52.42 **	26.43	73.03 **	65.58 **	35.84 *	73.92 **	66.53 **	75.12 **	81.21 **	103.18 **
Driage (%)	MPH	−5.79 **	−12.94 **	2.32	1.88	−1.93	−14.89 **	6.21 **	−3.91 **	10.13 **	7.29 **
	BPH	−9.45 **	−18.07 **	0.54	0.57	−4.06 **	−16.78 **	0.83	−8.04 **	2.4	4.10 **
Seed yield per fruit (g)	MPH	−13.56 **	−8.00 **	−1.71 *	−64.64 **	−0.42	6.15 **	12.53 **	−7.91 **	34.28 **	31.41 **
	BPH	−18.73 **	−9.61 **	−9.05 **	−72.40 **	−4.78 **	−7.17 **	−15.94 **	−16.16 **	3.49 **	9.04 **
Capsaicin (%)	MPH	64.12 **	82.03 **	66.79 **	56.46 **	88.80 **	62.93 **	69.06 **	72.33 **	62.93 **	60.45 **
	BPH	60.45 **	73.88 **	64.93 **	54.74 **	84.38 **	61.07 **	63.50 **	66.41 **	54.01 **	56.93 **
Oleoresin (%)	MPH	−15.79 **	26.58 **	23.68 **	24.71 **	−12.33 **	17.14 **	41.77 **	45.21 **	14.63 **	37.47 **
	BPH	−21.95 **	21.95 **	14.63 **	20.45 **	−15.79 **	17.14 **	27.27 **	39.47 **	6.82	23.41 **
Ascorbic acid (mg/100 g)	MPH	26.89 **	23.60 **	6.21 **	−3.17 **	18.26 **	14.43 **	26.96 **	9.35 **	21.95 **	24.23 **
	BPH	25.69 **	23.56 **	4.66 **	−3.64 **	17.10 **	13.83 **	26.37 **	7.71 **	21.32 **	23.00 **
Colour value (ASTA units)	MPH	11.20 **	18.60 **	22.42 **	24.53 **	−1.52 **	−5.24 **	24.03 **	−15.11 **	2.25 **	3.90 **
	BPH	1.84 **	2.92 **	5.55 **	6.80 **	−7.21 **	−11.36 **	15.35 **	−15.76 **	0.84 **	3.25 **

** and * indicate significant at *p* < 0.01 and *p* < 0.05, respectively.

**Table 6:** Details of parents used for hybridisation.

Name of Parents	Accession Number	Source *
P_1_	EC-391083	NBPGR, Hyderabad
P_2_	EC-596920	NBPGR, Hyderabad
P_3_	EC-596940	NBPGR, Hyderabad
P_4_	EC-599969	NBPGR, Hyderabad
P_5_	Dharwad local-2	UAS, Bangalore

* National Bureau of Plant Genetic Resources (NBPGR), Hyderabad, and the University of Agricultural Science (UAS), Dharwad, respectively.

**Table 7:** Weather parameters during the first and second crop season.

Month	Average Low Temperature (°C)	Average High Temperature (°C)	Average Precipitation (mm)
Season 1			
May	27	35	391
June	24	30	576
July	23	29	391
August	23	30	367
September	24	30	417
Season 2			
October	24	30	467
November	23	31	223
December	21	31	47
January	20	31	32
February	22	32	26
