# Correction: C. S. et al. Diallel Analysis of Chilli Pepper (*Capsicum annuum* L.) Genotypes for Morphological and Fruit Biochemical Traits. *Plants* 2020, *9*, 1

**DOI:** 10.3390/plants15132042

**Published:** 2026-07-01

**Authors:** Aiswarya C. S., Vijeth S, Sreelathakumary I, Prashant Kaushik

**Affiliations:** 1 Department of Vegetable Science, College of Agriculture, Kerala Agricultural University, Thrissur 680656, Kerala, India; aiswaryachandrasenannair2@gmail.com (A.C.S.); vijet129@gmail.com (V.S.); sreelatha.i@kau.in (S.I.); 2 Nagano University, 1088 Komaki, Ueda 386-0031, Nagano, Japan

In the publication [1], there was an error regarding the affiliation of Prashant Kaushik. The affiliation “Instituto de Conservación y Mejora de la Agrodiversidad Valenciana, Universitat Politècnica de València, 46022 Valencia, Spain” has been removed, as per the institution’s request. The authors state that the scientific conclusions are unaffected. This correction was approved by the Academic Editor. The original publication has also been updated.
